# Supplementary material for: Concurrent circulation of dengue serotype 1, 2 and 3 among acute febrile patients in Cameroon
Source: PLoS Negl Trop Dis. 2021 Oct 25;15(10):e0009860. doi: 10.1371/journal.pntd.0009860 (PMC8568189; doi:10.1371/journal.pntd.0009860)
Supplement: Attachment — Submitted filename: Responses to the reviewers.docx [file pntd.0009860.s001.docx]

**"Concurrent circulation of dengue serotype 1, 2 and 3 among acute febrile patients in Cameroon"** (PNTD-D-21-00715), by TCHETGNA et al

Dear Editor,

On behalf of my co-authors, I would like to thank the reviewers for the critical reading of our manuscript. We have considered their requests and made most of the changes suggested. Please, find below the point-by-point answers to reviewers, indicating which changes have been made, and where they have been inserted in the text (tracked changes in the revised version).

Sincerely yours,

Dr SIMO TCHETGNA

**Response to reviewer 1.**

**1.** Reviewer #1: The study aims to characterize the contribution of DENV to acute febrile illness in Douala, Cameroon. The authors obtained 320 samples from symptomatic patients and were able to detect DENV in 41 samples. The positive samples were serotyped by RT-qPCR and subsequently submitted to envelope gene sequencing. Maximum likelihood analysis was performed to identify the genotypes of DENV1, DENV2 and DENV3 circulating in Douala, Cameroon. The authors concluded that the simultaneous occurrence of three DENV serotypes in Douala reveals that the virus is a serious public health threat for Cameroon.

**Response**

Thank you for this comment

**2.** Reviewer #1: Some inconsistencies were observed in Results section; therefore, authors should clarify or rectify their statement in the text:Table 2: in the age column, the sum of positive dengue patients is not 41:

-    6 to 15 age line: the sum of positive and negative dengue cases is 48, but only 7 positive and 40 negative cases are listed

-    25 to 45 age line: the sum of positive and negative dengue cases is 103, but only 16 positive and 86 negative cases are listed

**Response**

Thank you for highlighting these mistakes. The modification has been done in the manuscript accordingly. Indeed, we have added a line for the data without any age value **See Table 2, page 11**

**3.** Lines 159-160: the number of dengue and malaria co-infections is inconsistent: 176 cases were co-infections with malaria, but only 41 patients tested positive for dengue. Is that accurate?

**Response**

Many thanks for this comment. It is an error in the abstract and author summary. Indeed, 55.6% of dengue cases (23/41) were co-infected with Malaria. This has been rectified in the manuscript. Please see the modifications at **lines 26 and 37**

**4.** Lines 177, 179, 185, 187 and 190: the names of the figures should be changed to Figure 2, 3 and 4 instead of Figure 2a, 2b and 2c.

**Response**

Thank you for your remark. The modifications have been done in the manuscript

178, 180, 186, 188, and 191

**5.** Figure 2, Figure 3 and Figure 4: ML trees were inferred with a number of dengue sequences different from the mentioned number of sequences generated in this study, for example: the authors generated 2 DENV1 sequences, but the ML tree shows only 1 sequence. The same was observed for DENV2 and DENV3. The authors should include all sequences generated in this study for phylogenetic analysis or make it clear why only some sequences were used.

**Response**

Thank for this remark. The number of sequences used for the ML trees have been included in the manuscript **(line133-134 and 301-302)** and the figure captions. Indeed, we were not able to sequence all the dengue positive samples obtained after the serotyping by real-time RT-PCR. The three were inferred with samples that were successfully sequenced.

**6**. Line 193: The authors mentioned that DENV3 strains from Cameroon might have evolved from South Africa strains. For clear understanding, the authors should provide the names of the countries they are referring to.

**Response**

Thank you for this input. Here we were referring to DENV-3 strains obtained in Madagascar the Comoros and Djibouti in 2010 and 2011 respectively. Please see **lines 195-196.**

**7.** Line 226: the ages of the patients co-infected with DENV2 and DENV3 are inconsistent with the information presented on f the Table 3.

**Response**

Thanks for this observation. There was a confusion with Ct values for the ages.. The right ages, contained in Table 3, have been included in the text. Please see in **line 228.**

**8.** Reviewer #1: The conclusions provided by the authors are sounded, but little is discussed about the limitations of the study. The authors discussed that their results are relevant for public health in Africa and how the monitoring of virus could help preventing dengue outbreaks.

**Response**

Thank you for your remark. Indeed, the study has some limitations including the fact that it is a cross-sectional study which was only done during one period of the year. A year-round study would have really spotted the period where the disease is more prevalent. Additionally, no follow-up of the patient or biological analysis has been done to be able to describe the clinical evolution of the disease, hence the outcome of the disease and possible haemorrhagic manifestations. Finally, we have not succeeded in sequencing all the viral strains obtained and we only sequenced a portion of the envelope gene. The sequencing of the whole genome of all the strains would have provided more insights into the genotypes and lineages that are active in the city as well as the genetic polymorphism of those strains. A paragraph was added in the manuscript accordingly. Please see **lines 280-285**.

**9.** Reviewer #1: Some inconsistencies were observed in Results section and related tables; therefore, authors should clarify or rectify their statement in the text.

**Response**

Thank You for this input. The inconsistencies have been corrected throughout the manuscript.

**-----------------------------------**

**Responses to reviewer 2**

**10.** Reviewer #2: The objectives are clear and well delineated for the study. The design is appropriate, however the sample size was low to compose four public hospitals. Even being from a poor country with a lack of surveillance. The low number of samples that were sequenced are unrepresentative. It is known that DENV1 is well established in the Aedes aegypti mosquito on several continents. Currently, dengue viruses have originated lineages within a genotype and this is very important to show the variability, especially for dengue viruses 1 and 2. It is important to emphasize that there are already many methodologies for obtaining low-cost and sensitive sequences that allow a broad analysis.

The target population is in agreement with the proposed objectives. Statistical tests are adequate, as well as molecular analysis. Ethical aspects are reported in the text.

**Response**

Thank you for this remark.

Concerning the sample size, it only includes the volunteers who have accepted to participate to the study. We have had a lot of refusal from patients meeting our inclusion criteria because the local population is not usually receptive to research involving blood collection, a situation that was intensified by the misinformation which have circulated during the early stages of the current COVID-19 pandemic. Additionally, to the latter, the frequentation level of the hospitals had significantly dropped at the time when we were doing the sample collection. All the positive samples obtained where not sequenced because of the low viral load (High Ct value) of some samples and also because we did not have the funding to go for more sensitive sequencing methodologies. Hence, we are really looking for those low-cost sequencing methods to obtain more sequences and performed a deeper analysis of dengue lineages. However, this article represents the first report of multiple dengue serotype occurrence in Cameroon. However, we have planned to go further in the analysis of our dengue strains to have a broader understanding of the evolution of this virus in the city.

**11.** Reviewer #2: The study proposed to determine the DENV serotypes and genotypes of dengue cases in four public hospitals, however a low number of positives was obtained by molecular biology. With regard to genetic sequencing, few sample sequences were obtained, this may be related to the sequencing methodology for genomic analysis used to determine the genotypes. There is an important reference in the study that malaria was found in more than 70% of cases and people are co-infected with two denv serotypes, such as denv2 and denv3.

The results are clear, with a lot of clinical information, which demonstrate good accuracy in data collection. This is relevant epidemiological information, as there is little data of this type in the study area, as endemic malaria makes it difficult to detect dengue cases. Studies of this type should be strengthened, as the clinical manifestations of dengue have caused serious conditions, such as dengue haemorrhagic fever and neurological alterations.

Figures are of good quality, but the table 2 needs improvement in presentation, I suggest a subdivision within it, to separate population demographic characteristics from clinical manifestations.

**Response**

Thank you for these remarks. The table 2 have been subdivided for more clarity. **See page 11**

**12.** Reviewer #2: The conclusion reports on the importance of arbovirus surveillance in urban areas in Africa, which is really important for public health. To improve viral surveillance, it is necessary to change the study algorithm, in order to have a closer phylogenetic profile of the genotypes, a greater number of sequences must be obtained, including using more sensitive methods, such as next-generation sequencing or at least larger partial sequences by Sanger.

At no time were the limitations of the study reported, such as difficulties with methodologies. Phylogenetic analyses are in agreement and provided important and reliable information, but for a more detailed study of genotypes it is necessary to increase the fragment size, as well as the number of sequences.

**Response**

That you for these remarks. We have included some limitations of our study in the manuscript concerning the size of the fragment sequenced and the number of sequences obtained. Please, see **lines 280-285**

**13.** Reviewer #2: Strength of the study:

1- Contribute to the knowledge of the situation of arboviruses in urban areas in Africa;

2- Identify circulating serotypes and genotypes;

3- Propose DENV surveillance in the study area, as three circulating serotypes were detected.

**Response**

Thanks for this good advice.

------------------------------------

**Response to reviewer 3**

**14.** Reviewer #3: The article proposed by SIMO TCHETGNA et al., acute febrile patients were presenting at hospitals between July and December 2020 for monitoring the circulation and co-circulation of DENV in Douala, Cameroon. Still, some details in the study queue should be taken into account:

The quantity of dataset samples is not representative of the phylogenetic inference analyses. I recommend the authors check the phylogenetic signal of the dataset before carrying out the phylogenetic reconstructions, considering that it is only the envelope region of the virus, requiring a more extensive set of data to represent the cohesive statements of the cohesive statements the analyses. It would be interesting for the authors to check the introduction of serotypes. It is not common to have three dengue serotypes circulating in a small region of a country. What were the criteria used for the sequencing of the samples, considering that of the 41 positives, only nine samples were sequenced, only 21% were sequenced? Was there sequencing of co-infection samples between serotypes 2 and 3? I believe that the results need to be better worked to accept the article in the newspaper, so I don't recommend the article in its format. the introduction of serotypes. It

**Response**

Thank you for this remark.

It is true that it is not common to have three serotypes circulating in a small region of a country however, this situation is increasingly reported in Africa ^1-3^ . These results were generated by using, three different RT-PCR with already published primers sets targeting different genomic portions (Two real-time PCR and an end-point PCR) to be able to detect dengue and its serotypes. Although, we only analyse the envelop gene, it is sufficient to differentiate the various serotypes and it has been extensively used for that purpose by various authors^3^^,^^4^. Additionally, the serotypes have also been differentiated by the analysis of the Capsid and 3’UTRs during the second real time RT-PCR. We have not sequenced all the positive sample for various reasons including the viral load (Ct value), the funding and the availability of raw sample. However, more sequencing analysis will be done with more samples and longer genomic fragments to properly infer the origin and introduction of theses serotypes

We have considered your suggestion to testing the phylogeny before the reconstruction and testing the introduction of the serotypes.

**Reference**

1	Konongoi, L. *et al.* Detection of dengue virus serotypes 1, 2 and 3 in selected regions of Kenya: 2011-2014. *Virol J* **13**, 182, (2016).

2	Chipwaza, B. *et al.* Occurrence of 4 Dengue Virus Serotypes and Chikungunya Virus in Kilombero Valley, Tanzania, During the Dengue Outbreak in 2018. *Open forum infectious diseases* **8**, ofaa626, (2021).

3	Caron, M. *et al.* First evidence of simultaneous circulation of three different dengue virus serotypes in Africa. *PLoS One* **8**, e78030, (2013).

4	Carreno, M. F. *et al.* Dengue in Santander State, Colombia: fluctuations in the prevalence of virus serotypes are linked to dengue incidence and genetic diversity of the circulating viruses. *Trop Med Int Health* **24**, 1400-1410, (2019).
